# Evaluation of temporomandibular disorders by magnetic resonance imaging

**DOI:** 10.1590/0100-3984.2019.52.3e2

**Published:** 2019

**Authors:** Natália R. Ferreira, Marcos F. DosSantos

**Affiliations:** 1 Laboratory of Cell Morphogenesis/Institute of Biomedical Sciences, and Graduate Program in Medicine (Radiology)/School of Medicine - Universidade Federal do Rio de Janeiro (UFRJ), Rio de Janeiro, RJ, Brazil.

Temporomandibular disorders (TMDs) are widely described as heterogeneous conditions affecting the temporomandibular joint (TMJ) and masticatory muscles. However, TMD is a highly generic term, referring to a group of disorders arising from the muscles (myogenic TMDs) or the joint (arthrogenic TMDs). The most common arthrogenic TMDs are anterior disc displacement of the articular discs and TMJ osteoarthritis. Each of those disorders has a complex pathophysiology and multiple risk factors, which interact in different ways in each individual^(^^1^^)^. In addition, pain chronicity, with its central mechanisms, such as neuroplasticity and central sensitization, as well as the participation of psychogenic factors, makes it especially challenging to gain a complete understanding of the pathophysiology of these disorders^(^^2^^)^.

The article "Changes in temporomandibular joint anatomy, changes in condylar translation, and their relationship with disc displacement: magnetic resonance imaging study" published in the previous issue of **Radiologia Brasileira**^(^^3^^)^ makes important contributions to the understanding of internal TMJ disorders, mainly due to the large number of joints studied. The authors demonstrated that changes indicative of osteoarthritis were more common on the articular surfaces of TMJs with anterior articular disc displacement than on those of TMJs presenting normal articular disc positioning, corroborating the findings of previous studies involving considerably smaller samples. The study of interest, conducted by Bedran et al.^(^^3^^)^, presents interesting data regarding the osseous alterations of the articular eminence and the articular fossa, findings that are highly relevant, considering that those anatomical sites are often neglected in the analysis of internal TMJ disorders on imaging examinations. However, despite the timely presentation of the data and the important contribution of that study to a better understanding of the internal disorders of the TMJ, the study design precludes any reliable inferences regarding temporal or causal relationships between or among the different types of internal TMJ disorders.

The causal relationship between osteoarthritis and anterior disc displacement of the TMJ is still not well understood. It has been suggested that a change in the position of the articular disc would lead to an increased mechanical load on the TMJ, thus contributing to the development of osteoarthritis. That theory is usually corroborated by data from studies demonstrating the association between the two disorders (osteoarthritis and anterior disc displacement of the TMJ). The study conducted by Roh et al.^(^^4^^)^ indicated that the risk of degenerative changes is 2.0 times higher in cases of anterior disc displacement with TMJ reduction and 4.4 times higher in cases of anterior disc displacement without TMJ reduction. Dias et al.^(^^5^^)^ demonstrated that degenerative changes are 8.25 times more likely to occur in cases of anterior disc displacement without TMJ reduction. However, other authors have suggested that bone alterations caused by TMJ osteoarthritis would lead to an incompatibility between the articular disc and the mandibular condyle, with consequent anterior disc displacement of the TMJ, which would also explain the higher prevalence of anterior disc displacement in degenerative TMJ disorders^(^^6^^)^. In addition, it has been hypothesized that osteoarthritis and anterior disc displacement of the TMJ represent distinct entities, given that there are cases of osteoarthritis in TMJs with normal disc position and cases of TMJs with anterior disc displacement and no bone changes. Another important issue that must be discussed is whether anterior disc displacement and TMJ osteoarthritis share the same risk factors, such as joint overload, which would also help explain the frequent concomitant presence of the two disorders in the same joint^(^^4^^)^.
